# Re-Evaluating the Progesterone Challenge Test as a Physiologic Marker of Endometrial Cancer Risk: A Systematic Review and Meta-Analysis

**DOI:** 10.3390/diagnostics16030378

**Published:** 2026-01-23

**Authors:** Rachel J. Woima, Derek S. Chiu, Elise Abi Khalil, Sabine El-Halabi, Andrea Neilson, Laurence Bernard, Jessica N. McAlpine, Aline Talhouk

**Affiliations:** 1 Department of Obstetrics and Gynecology, Faculty of Medicine, University of British Columbia, Vancouver, BC V6T 1Z3, Canada; rjwoima@uwaterloo.ca (R.J.W.); derek.chiu@ubc.ca (D.S.C.); elise.abikhalil@ubc.ca (E.A.K.); halabis2@student.ubc.ca (S.E.-H.); andrea.neilson@ubc.ca (A.N.); jessica.mcalpine@vch.ca (J.N.M.); 2 Department of Gynecologic Oncology, McGill University Health Centre (MUHC), McGill University, Montréal, QC H4A 0B1, Canada; laurence.bernard@medportal.ca

**Keywords:** progesterone challenge test, endometrial cancer, endometrial hyperplasia, diagnostic accuracy, physiologic biomarker, risk stratification, postmenopausal women, estrogen-driven carcinogenesis, preventive gynecology, hormone responsiveness

## Abstract

**Background/Objectives**: With the rising incidence of obesity-related endometrial cancer, there is renewed interest in physiologic, low-cost approaches to identify women with hormonally active endometrium who may benefit from early preventive interventions. The progesterone challenge test (PCT), an established clinical tool for evaluating amenorrhea, has been previously proposed as a method to detect endometrial pathology. This study systematically evaluated the diagnostic accuracy of the PCT for detecting endometrial hyperplasia, intraepithelial neoplasia, and carcinoma in asymptomatic postmenopausal women to determine its potential role as a physiologic marker of endometrial cancer risk. **Methods**: A systematic review and meta-analysis were conducted following PRISMA-DTA guidelines. MEDLINE, EMBASE, EBM Reviews, and CINAHL were searched from inception to 20 January 2025, along with ClinicalTrials.gov and grey literature. Eligible studies prospectively evaluated the PCT with endometrial biopsy as the reference standard. Data extraction and risk-of-bias assessment were performed in duplicate. Risk of bias was assessed using QUADAS-2. Pooled sensitivity, specificity, and predictive values were estimated using hierarchical summary receiver operating characteristic models. **Results**: Nineteen studies (n = 3902) met the inclusion criteria. The pooled sensitivity and specificity of the PCT for detecting endometrial pathology were 95% (95% CI 86–100%) and 87% (76–96%), respectively. The positive predictive value was 32% (95% CI, 16–50%) and the negative predictive value was 100% (100–100%). When endometrial proliferation was included in the target condition, sensitivity decreased to 82%, but positive predictive value increased to 70%. **Conclusions**: The PCT shows high diagnostic accuracy for identifying estrogen-driven endometrial pathology in asymptomatic postmenopausal women. Re-evaluating this simple, physiologic test as a functional risk-stratification tool could inform precision prevention strategies for endometrial cancer.

## 1. Introduction

Endometrial cancer (EC) is the most common gynecologic cancer in high-income countries [1], with an estimated 417,000 new cases and 97,000 deaths in 2022 [1]. EC continues to see a global increase in incidence and mortality [2,3], a trend driven in part by rising obesity rates, the strongest modifiable risk factor for EC [4,5]. After menopause, endogenous hormone production ceases, but adipose tissue can continue to produce hormones through the aromatization of androgens [6,7]. This results in persistent, unopposed estrogenic stimulation of the endometrium, promoting continuous proliferation that can progress to hyperplasia, endometrial intraepithelial neoplasia (EIN), and ultimately estrogen-driven endometrioid endometrial carcinoma (EEC), which accounts for over 80% of all EC diagnoses [8,9]. Importantly, not all women with obesity are at equal risk of EC; risk depends on the degree of estrogenic stimulation within the endometrium, which cannot be inferred from body mass index alone. Identifying those with a hormonally active endometrium is therefore critical for guiding preventive strategies.

Although a proliferative endometrium is not currently classified as pathologic, it represents a biologically active precursor state. Women with a proliferative endometrium are at a four-fold increased risk of hyperplasia or cancer over 10 years, compared to those with an atrophic endometrium [10]. Risk-reducing measures, including progestogen therapy (e.g., levonorgestrel intrauterine device), weight loss, and lifestyle modification, can reverse early estrogen-dependent changes and even treat early-stage disease [11,12,13,14,15]. However, these interventions are not cost-effective at the population level [16,17,18]. There is currently no screening recommendation for EC in asymptomatic people; in high-risk women with hereditary Lynch Syndrome, transvaginal ultrasound and endometrial sampling may be considered, but these approaches are invasive and resource-intensive [19]. Detecting estrogenic endometrial activity could create an opportunity for timely prevention.

The progesterone challenge test (PCT) is a short course of progestogen (5–10 days) followed by observation for withdrawal bleeding. In postmenopausal women, a positive result (bleeding) indicates the presence of circulating estrogen and a responsive endometrium, suggesting underlying proliferation or pathology. It was first proposed as a screening tool for EC in the 1980s [20] and remains widely in use in reproductive endocrinology to assess hormonal status in amenorrheic patients [21]. Given its low cost, safety, and scalability, the test could be particularly valuable in guiding prevention in women with obesity, a group at highest risk for estrogen-driven EC and for whom hysterectomy, the first line of therapy for EC, carries increased surgical risks [22]. Despite several individual studies evaluating its diagnostic performance, the test has not been systematically reviewed or quantitatively synthesized. Re-evaluating the PCT through a diagnostic accuracy lens and synthesizing the existing evidence will consolidate historical data.

This work aims to analyze the PCT’s diagnostic performance and establish its potential role as a physiologic marker to identify postmenopausal women with persistent estrogenic endometrial activity who could benefit from targeted progestin therapy or closer surveillance. Specifically, we systematically reviewed and meta-analyzed published evidence to evaluate the test’s sensitivity, specificity, positive and negative predictive values for detecting endometrial pathology, with or without the inclusion of endometrial proliferation as part of the target condition.

## 2. Methods

We followed PRISMA-DTA (Preferred Reporting Items for Systematic Reviews and Meta-analyses for Diagnostic Test Accuracy) guidelines [23,24]. A protocol was registered in PROSPERO (CRD42023272301) on 8 August 2023.

*Eligibility Criteria*: We included prospective studies evaluating the PCT in postmenopausal women with an intact uterus and no abnormal uterine bleeding. Eligible designs included cohort and case–control studies published in English or French prior to April 2025. Studies had to assess the PCT’s ability to detect endometrial pathology, defined as hyperplasia (with or without atypia), EIN, or endometrial carcinoma. A secondary analysis assessed diagnostic accuracy by adding endometrial proliferation to the target condition.

We included studies using any variations of the PCT (index test): a short course (<15 days) of oral or injectable progestogen followed by observation for withdrawal bleeding. The reference standard was endometrial sampling via biopsy (curettage, aspiration, or hysteroscopy). Cytology-based assessments were excluded. To be eligible, studies had to report the total number of PCTs administered and the number of positive results (+PCT)

*Search Strategy*: We searched MEDLINE (Ovid), EMBASE (Ovid), EBM Reviews (Cochrane), Web of Science, and CINAHL (EBSCO) using terms related to menopause, progesterone, and endometrial pathology from inception to 20 January 2025. An initial search was completed on 20 January 2023, and was subsequently updated on 10 April 2025, to ensure inclusion of the most recent literature. This update deviates from the registered protocol. We also searched ClinicalTrials.gov and grey literature sources, and manually reviewed the reference lists of included studies. The search strategy, developed in collaboration with a health sciences librarian, is detailed in Supplementary File S1.

*Study Selection and Data Extraction*: Search results were imported into Covidence (https://www.covidence.org/ (accessed on 8 January 2025)) for deduplication and screening. Two authors independently screened abstracts and full texts, extracted data, and resolved discrepancies with a third reviewer. We attempted to contact authors when full-text articles were not available for review. Extracted variables included study setting, population characteristics, PCT protocol, sampling method, and histological outcomes. Risk populations were defined based on inclusion criteria (e.g., obesity, diabetes, prior tamoxifen use, or increased endometrial thickness).

Outcomes were categorized based on histology (Table S1). Diagnostic classifications, true positives (TP), false positives (FP), true negatives (TN) and false negatives (FN), were extracted for both primary and secondary target conditions.

Risk of Bias: Two reviewers independently assessed study quality by adapting the QUADAS-2 tool (Quality Assessment of Diagnostic Accuracy Studies) [25] to include items on the definition and application of the PCT. Full criteria are available in Supplementary File S2.

Data Synthesis and Analysis: The pooled PCT positivity rate was estimated using a random-effects model with an arcsine variance-stabilizing transformation [26,27]. Meta-regression evaluated associations between positivity rates and PCT characteristics (dosage, progestogen type, number of days on progestogen, observation window, and timing of endometrial sampling). The meta-regression was performed using an intercept-parameterized model for hypothesis testing (omnibus p-values) and a no-intercept parameterization to obtain level-specific point estimates and confidence intervals. Forest plots were generated using a random-effects model with a common τ^2^ (between-study heterogeneity) to ensure consistency with the meta-regression parameterization. Miss rates were also calculated, and positivity rates were compared with disease prevalence in studies that performed biopsies.

Sensitivity and specificity were pooled using a hierarchical summary receiver operating characteristic (HSROC) model [28,29]. A bivariate model was used for comparison [30]. Positive Predictive Value (PPV) and Negative Predictive Value (NPV) were also computed and pooled. Studies that sampled only +PCT patients and reported TP and FP values were included only in the PPV analysis. We computed PPV and NPV across a range of hypothesized disease prevalence (2% to 50%), using the HSROC-estimated pooled sensitivity and specificity. Computations used the R packages HSROC (version 2.1.9) and meta (version 4.3.3) [31,32].

Core Outcome Sets: No core outcome sets specific to endometrial cancer screening or diagnostic accuracy studies were available or applicable at the time of this review.

Patient and Public Involvement: Patients and the public were not involved in the design, conduct, or reporting of this study.

## 3. Results

### 3.1. Study Characteristics

**Figure 1:**
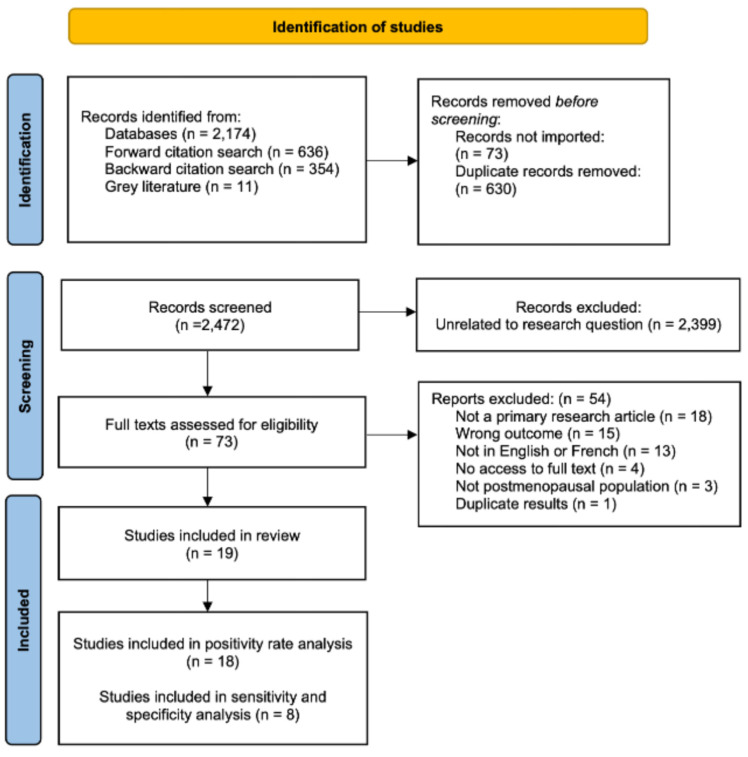
PRISMA Flowchart.

After de-duplication, 2472 records were identified. Following title and abstract screening, 2399 articles were excluded because they were not relevant to the research question. Full texts of 73 articles were assessed for eligibility; 54 were excluded for not being primary research article, not measuring positive PCT rate or diagnostic accuracy, not being done in postmenopausal participants, not being in English or French and not providing access to the full text. Detailed exclusion rational for each study is Supplementary Table S2. A total of 19 studies met the inclusion criteria and were retained for data extraction and synthesis. The included studies comprised 3902 post-menopausal participants. Study characteristics, along with extracted diagnostic accuracy data, are summarized in Table S3. The PRISMA flow diagram outlining the study selection process is shown in Figure 1.

The studies were conducted across nine countries, primarily in Europe and North America, and most were published before 2010. Six studies (n = 329) were classified as ‘high-risk populations’ based on selection for known EC risk factors [33], such as endometrial thickness from transvaginal sonography greater than >5 mm [34,35,36] or Tamoxifen use [37,38]. The remaining 13 studies (n = 3573) represented general postmenopausal populations. Endometrial sampling was performed on all participants, regardless of PCT result, in seven studies (n = 530) [37,38,39,40,41,42,43], and one study biopsied all +PCT patients (n = 14) and a subset of -PCT patients (n = 30) [33]. Nine studies performed biopsies only among participants with +PCT (n = 379) [33,34,35,36,44,45,46,47,48,49]. Two studies reported performing biopsies without providing histological results [50,51]. Participants with failed [33,41] or infeasible biopsies [37,39,45] were excluded from diagnostic accuracy calculations. Table 1 presents the full study characteristics.

### 3.2. Risk of Bias

Across studies, 21% were rated as high risk and 63% as unclear for patient selection bias due to incomplete enrolment details. The risk of bias related to PCT administration was low in 53% of studies and unclear in 42%. Blinding of histopathological assessment was infrequently reported. Several studies explicitly recruited high-risk women (e.g., tamoxifen users or women with increased endometrial thickness), while others did not report baseline risk factors, making it difficult to determine how representative the study populations were. Consecutive or random sampling was rarely reported, raising concerns about potential selection bias. Notably, time since menopause was almost never documented. Protocols for the progestogen dose and duration varied widely, and some studies did not provide sufficient detail to determine whether the test was applied consistently across participants. Flow and timing bias were generally low across studies, and no major concerns were raised about applicability. Risk of bias assessments are summarized in Figures S2 and S3.

### 3.3. PCT Positivity Rates, Miss Rates and Prevalence

**Figure 2:**
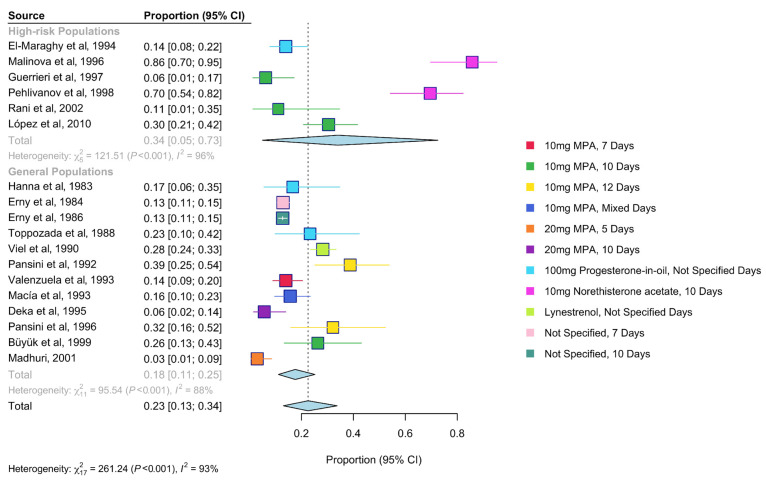
Forest plot of pooled total exposure positivity rate. The estimated positivity rate for each study with 95% confidence intervals. Studies are presented in publication order, coloured by progesterone type and dosage used in the PCT. High-risk and general populations were pooled separately and jointly [33,34,35,36,37,38,39,40,41,42,44,45,46,47,48,49,50,51]. The blue diamond represents the pooled effect estimate, with its width indicating the 95% confidence interval.

The one case–control study [43] was excluded from the positivity rate analysis. Among the remaining 18 studies, the +PCT rate was 23% (95% Confidence Interval (CI), 13–34%) (Figure 2). High-risk populations had a higher +PCT rate of 34% (95% CI, 5–73%) compared to 18% (95% CI, 11–25%) in the general population, although this was not statistically significant (p-value = 0.14). In contrast, PCT dose and the type of progestogen were significantly associated with the +PCT rate (p-value < 0.01). Norethisterone acetate was associated with the highest positivity rates (70% and 86%), while the lowest was observed with 20 mg medroxyprogesterone acetate (MPA) administered for 5 days (3%, 95% CI: 1–9%). PCT timing and monitoring duration were not significantly associated with positivity rates (Table 2). Disease prevalence was not consistently higher in high-risk studies compared to the general population (Figure S3).

A total of 954 endometrial biopsies were reported across 17 included studies. Table 3 summarizes the distribution of histologic findings in reported biopsies. We reported pathology findings for all biopsies from +PCT individuals and also restricted the analysis to the eight studies that performed 560 biopsies in both test groups, irrespective of PCT results. Across all studies, atrophic endometrium was the most common finding, followed by benign polyps or fibroids, proliferative endometrium, and non-atypical hyperplasia. Hyperplasia with and without atypia and endometrial carcinoma were almost never identified in negative PCT participants in the subset of studies that biopsied both.

### 3.4. Sensitivity and Specificity

Eight studies that biopsied all participants were included in the diagnostic accuracy analysis. For the primary outcome (endometrial pathology), pooled sensitivity was 95% (95% CI, 86–100%) and specificity was 87% (95% CI, 76–96%). When endometrial proliferation was included in the target condition, sensitivity decreased to 82% (95% CI, 58–99%) and specificity increased to 94% (95% CI, 83–100%).

**Figure 3:**
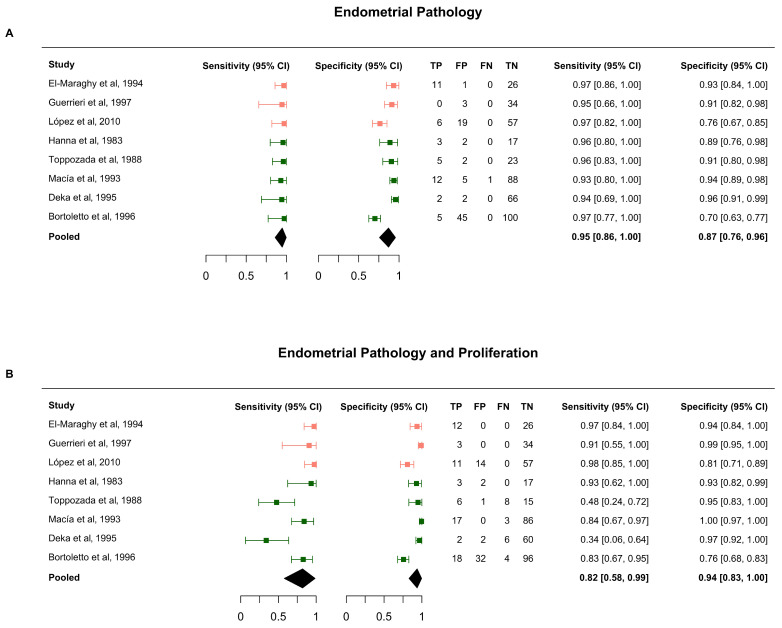
Forest plots of sensitivity and specificity from the HSROC model for (**A**) endometrial pathology and (**B**) endometrial pathology and proliferation. Pooled sensitivity and specificity estimates are reported for each study, along with 95% confidence intervals. The studies are ordered by date. High-risk study populations are in pink, and general population studies are in dark green. The top panel reports the sensitivity and specificity of PCT for detecting endometrial pathology, and the bottom panel shows endometrial proliferation as part of the target condition [33,37,38,39,40,41,42,43]. TP = true positive, TN = true negative, FP = false positive, and FN = false negative. The diamond represents the pooled effect estimate, with its width indicating the 95% confidence interval.

Results were consistent across HSROC and bivariate models (Figure 3 and Figure S4). The overall pooled miss rate was low (1%, 95% CI, 0–4%), but increased to 13% (95% CI, 0–43%) when proliferation was included in the target condition (Figure S5).

### 3.5. Predictive Values

**Figure 4:**
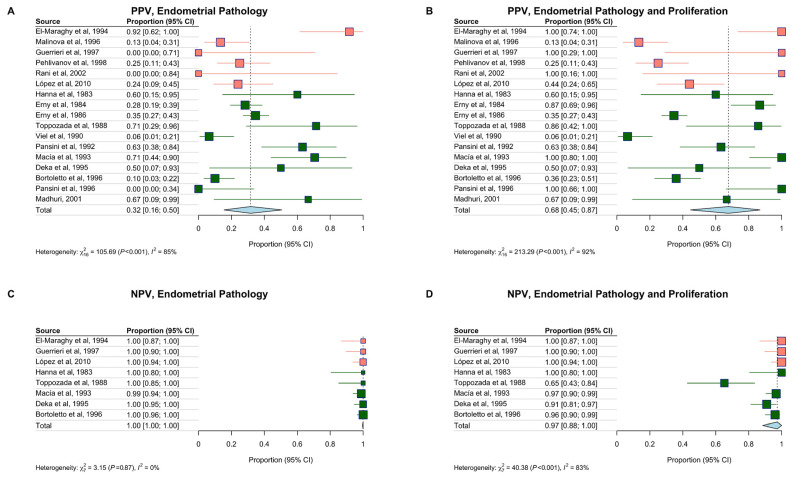
Positive Predictive Value (**A**,**B**) and Negative Predictive Value (**C**,**D**) of the PCT in detecting endometrial pathology (**A**,**C**) and endometrial pathology and proliferation (**B**,**D**). The pooled PPV and NPV estimates are reported for each study with 95% confidence intervals. The studies are ordered by publication year within each population. General population studies are in dark green, and high-risk study populations are in pink [33,34,35,36,37,38,39,40,41,42,43,44,45,46,47,48,49]. The diamond represents the pooled effect estimate, with its width indicating the 95% confidence interval.

PPV was calculated in 17 studies and was 32% (95% CI, 16–50%) for endometrial pathology. NPV was based on 8 studies and was 100% (95% CI, 100–100%). When endometrial proliferation was included in the target condition, PPV increased to 68% (95% CI, 45–87%), and NPV slightly decreased to 97% (95% CI, 88–100%) (Figure 4). Predictive values were modelled across a range of prevalence estimates. PPV exceeded 50% when the prevalence of pathology was above 15% (Figure S6), while NPV remained consistently high regardless of prevalence.

## 4. Discussion

This systematic review and meta-analysis consolidates findings from 19 previously published studies, including 3902 participants, that investigated the diagnostic accuracy of PCT.

Results demonstrate that differences in the observed PCT positivity rate were associated with the progestogen regimen. Norethisterone acetate yielded higher +PCT rates, possibly due to its partial estrogenic activity [52,53], structural similarity to testosterone, and metabolic conversion to ethinylestradiol [52]. Studies with short progestogen courses (<7 days) or low doses yielded lower response rates, underscoring the importance of both dosage and duration [49].

Our analysis found the PCT to be a sensitive and specific test for detecting estrogen-driven endometrial pathology in asymptomatic postmenopausal women. The pooled sensitivity and specificity were 95% and 87%, respectively, with an exceptionally high negative predictive value. Even though our analysis excluded a number of non-English/French studies and conference abstracts, the reported diagnostic metrics from those sources were broadly similar (sensitivity 82–100%, specificity 78–97%) [54,55,56,57].

In the 435 PCT-negative biopsies from the 8 studies that biopsied all participants, only 1 EIN was missed, indicating a strong NPV for pathologic diagnoses. PPV was estimated at ~30% but varied greatly across studies. This is because PPV largely depends on disease prevalence, which varies across populations. Including endometrial proliferation as part of the target condition increased the positive predictive value to 70% but modestly reduced sensitivity. While a proliferative endometrium is not currently treated or monitored clinically, it may represent an early, reversible stage of endometrial carcinogenesis [10]. Several studies also noted that the PCT identified other estrogen-responsive conditions, including endometrial polyps, which themselves may be associated with an elevated risk of endometrial cancer [58].

This is the first study to synthesize diagnostic accuracy data for the PCT across diverse populations and protocols. A key strength is the comprehensive search strategy that included grey literature and was developed with input from a medical librarian. Data extraction and risk-of-bias assessments were performed in duplicate, and appropriate hierarchical models were used for the meta-analysis. Although the studies included in this review were conducted in earlier decades, their mechanistic basis remains biologically relevant, since the physiologic response of the endometrium to progesterone challenge has not changed over time. However, it is important to note the inherent limitations due to heterogeneity across study settings. Some studies lacked detailed reporting of blinding, verification, or risk-stratification criteria. Definitions of obesity, endometrial thickness, and tamoxifen exposure varied substantially, limiting comparability. For example, obesity was not consistently defined by a BMI cut-off [7]. Similarly, different thresholds of endometrial thickness were used to assess risk in vaginal ultrasounds. Tamoxifen exposure thresholds ranged from 6 months to 2 years [37,38], even though EC risk is associated with the duration of Tamoxifen exposure [59]. Data on time since menopause were also frequently lacking, despite its known influence on bleeding likelihood, as withdrawal bleeding is more common in the early postmenopausal years when the endometrium may remain hormonally active. While several studies explicitly enrolled high-risk participants, others did not clearly specify this in their inclusion criteria but were likely biased toward higher-risk clinical populations. As a result, the pooled estimates of diagnostic accuracy should be interpreted as reflecting selectively enriched high-risk cohorts rather than a general postmenopausal population. The lack of long-term follow-up data prevents conclusions regarding progression or outcomes. Another practical limitation is that false-negative PCT results may occur in women with intrauterine or cervical adhesions, or in those with impaired endometrial shedding, where progesterone withdrawal bleeding cannot manifest despite underlying pathology [21]. False positives observed in atrophic endometria could be due to focal lesions missed during sampling or to breakthrough bleeding secondary to progestogen exposure [60,61,62]. The diagnostic performance estimates in this meta-analysis primarily reflect estrogen-driven endometrioid pathology, as non-endometrioid endometrial cancers were infrequently reported or not stratified by histology in the included studies and are not expected to reliably respond to the PCT.

Despite these limitations, diagnostic performance estimates were remarkably consistent across studies, supporting the robustness of the physiologic link between progesterone responsiveness and estrogen-driven endometrial pathology.

The PCT has long been used in gynecologic practice for evaluating amenorrhea. Still, it has not been incorporated into contemporary clinical guidelines or routinely used to guide EC screening or diagnosis. Its limited clinical uptake in this context likely reflects historical concerns surrounding progestogen therapy following the Women’s Health Initiative, which saw a decline in hormonal prescriptions overall [63]. The doses and durations used in PCT protocols are far below those associated with any adverse breast or cardiovascular outcomes and are unlikely to carry measurable long-term risk. Modern diagnostic pathways rightfully favour imaging modalities accompanied by endometrial sampling, as they allow immediate interpretation. These approaches are not currently recommended for screening asymptomatic populations, where the false-positive rate of ultrasound could exceed 90% depending on the thickness cutoff [64,65]. Moreover, endometrial thickness detected by transvaginal ultrasound does not necessarily reflect hormonal activity. Similarly, endometrial sampling is invasive and would likely not be acceptable or cost-effective in the general population.

Identifying women at-risk for endometrial pathology with hormonally active endometrium provides a physiologic basis for targeting prevention interventions. Withdrawal bleeding following the PCT can signal estrogen priming and functional progesterone receptors. The PCT may therefore serve as a non-invasive functional biomarker to guide asymptomatic postmenopausal women to interventions, such as levonorgestrel intrauterine devices (LNG-IUD), before neoplastic changes occur. The PCT compares favourably with other gynecological screening modalities. Its positive and negative predictive values are comparable to those of cervical cytology or HPV testing for cervical cancer screening (PPV: ~6–7% and NPV > 99%) [66]. These results reinforce the biological plausibility of the test and establish a solid empirical basis for its contemporary re-evaluation to guide prevention through standardized protocols and prospective validation. Finally, although most estrogen-driven endometrial neoplasms arise in the setting of a proliferative endometrium, a subset of endometrial cancers, particularly those with non-endometrioid histology, may develop in an atrophic endometrium, in which case progesterone withdrawal bleeding may not occur despite underlying malignancy. Accordingly, the PCT is not expected to identify all ECs and should be viewed as a functional marker of estrogen-responsive endometrium rather than a universal screening tool.

## 5. Conclusions

The PCT is a sensitive, specific, and low-burden tool for detecting estrogen-responsive endometrial pathology in postmenopausal women. Conceptually, the PCT could be explored as a periodic functional assessment in postmenopausal women at elevated risk for estrogen-driven endometrial pathology (e.g., high BMI or prolonged tamoxifen exposure), where a positive result would prompt targeted ultrasound or biopsy and help direct hormonal prevention (e.g., LNG-IUD) toward individuals with demonstrable hormonal responsiveness. In contrast, a negative result might support continued surveillance and repeat PCT testing every 2–3 years, without immediate action. Targeting preventive interventions to those most likely to benefit would improve their cost-effectiveness and facilitate their uptake. Given that most endometrial cancers are estrogen-dependent and obesity-associated, reintroducing the PCT as a physiologic risk-stratification test may help close a major gap in preventive gynecologic care.

For future clinical investigations, a standardized regimen, such as 10 mg MPA daily for 10 days, should be adopted to ensure test consistency. Finally, the PCT only detects estrogen-related ECs. These account for most EC patients and are typically associated with obesity. Non-endometrioid ECs are not estrogen-dependent and, as such, would not be detectable through PCT.

Key future research priorities should include establishing predictive value and cost-effectiveness in contemporary cohorts, assessing patient acceptability and the feasibility of implementation in primary and specialist care (NCT05651282), and linking PCT response to longitudinal clinical outcomes, including regression of hyperplasia and incident carcinoma. Further research should also determine the optimal frequency and interval for repeating the PCT (e.g., annually or biennially) to balance early detection with practicality and cost-effectiveness. These efforts could determine whether the PCT has a role as a modern, physiologic biomarker for precision prevention of endometrial cancer.

## Figures and Tables

**Table 1:** Study characteristics. Characteristics of reviewed studies, including population risk group, country of origin, PCT protocol (duration, dose, agent), monitoring window and biopsy timing.

Characteristic	Studies = 19	N = 3902
Population		
High-risk Populations	6 (32%)	329 (8.4%)
General Populations	13 (68%)	3573 (91.6%)
Country		
Brazil	1 (5.3%)	150 (3.8%)
Bulgaria	2 (11%)	81 (2.1%)
Egypt	2 (11%)	130 (3.3%)
France	3 (16%)	2800 (71.8%)
India	3 (16%)	188 (4.8%)
Italy	2 (11%)	77 (2.0%)
Spain	3 (16%)	360 (9.2%)
Turkey	1 (5.3%)	38 (1%)
United States	2 (11%)	78 (2%)
PCT timing (days)		
5	1 (5.3%)	100 (2.6%)
7	2 (11%)	984 (25.2%)
10	9 (47%)	2120 (54.3%)
12	2 (11%)	77 (2%)
Mixed	1 (5.3%)	121 (3.1%)
Not specified	4 (21%)	500 (12.8%)
PCT dose/day		
10 mg MPA	9 (47%)	691 (17.7%)
20 mg MPA	2 (11%)	170 (4.4%)
100 mg Progesterone-in-oil	3 (16%)	160 (4.1%)
10 mg Norethisterone acetate	2 (11%)	81 (2.1%)
Lynestrenol	1 (5.3%)	340 (8.7%)
Not Specified/Mixed	2 (11%)	2460 (63%)
Monitor Window		
5 days	1 (5%)	38 (1%)
10 days	6 (32%)	276 (7.1%)
14 days	4 (21%)	260 (6.7%)
30 days	1 (5.3%)	82 (2.1%)
25–30 days	1 (5.3%)	121 (3.1%)
Any number of days	3 (16%)	325 (8.3%)
Not specified	3 (16%)	2800 (71.8%)
Timing of endometrial biopsy *		
Prior to the PCT	6 (35.3%)	434 (45.5%)
After the PCT	11 (64.7%)	520 (54.5%)

* n (%) from only 17 studies that reported biopsy results in 954 participants.

**Table 2:** Meta-regression results evaluating the effect of PCT regimen and study characteristics on positivity rate. Estimates with corresponding 95% confidence intervals are presented. Bolded p-values indicate statistically significant associations.

Variable	Level	+PCT (95% CI)	p-Value
Population	High-risk Populations (Reference)	34% (16%, 55%)	0.14
	General Populations	18% (8%, 30%)	
PCT timing	5 (Reference)	3% (0%, 46%)	0.69
(days)	7	13% (0%, 49%)	
	10	28% (12%, 48%)	
	12	36% (5%, 76%)	
	Mixed	16% (0%, 69%)	
	Not specified	20% (3%, 47%)	
PCT	10 mg MPA (Reference)	21% (14%, 29%)	**<0.01**
dose/day	20 mg MPA	4% (0%, 14%)	
	100 mg Progesterone-in-oil	17% (7%, 31%)	
	10 mg Norethisterone acetate	78% (60%, 91%)	
	Lynestrenol	28% (10%, 51%)	
	Not Specified	13% (4%, 25%)	
Monitor	5 (Reference)	26% (0%, 83%)	0.70
	10	37% (16%, 61%)	
	14	13% (0%, 38%)	
	30	30% (0%, 85%)	
	25–30	16% (0%, 70%)	
	Any	13% (0%, 51%)	
	Not specified	17% (1%, 48%)	
Biopsy	Prior (Reference)	15% (3%, 33%)	0.24
timing	After	27% (15%, 41%)	

**Table 3:** Distribution of endometrial pathologies among +PCT and -PCT biopsies across all included studies. The first column summarizes pathology findings for all biopsied +PCT across the 17 studies that reported biopsy results for this group, including studies that biopsied only +PCT participants. The second and third columns present pathology findings from the 8 studies that performed biopsies in both +PCT and at least a subset of PCT women, providing a less biased representation of pathology distribution in the two groups.

	All	Positive/Negative	
Condition	+PCT	+PCT	-PCT
Endometrial Carcinoma	10 (1.9%)	1 (0.8%)	0 (0%)
Atypical Hyperplasia or EIN	22 (4.2%)	18 (14.4%)	1 (0.2%)
Hyperplasia Without Atypia	120 (23.1%)	25 (20.0%)	0 (0%)
Proliferative	39 (7.5%)	28 (22.4%)	20 (4.6%)
Benign Conditions (Fibroid, Polyps)	96 (18.5%)	16 (12.8%)	16 (3.7%)
Atrophic Endometrium	215 (41.4%)	32 (25.6%)	355 (81.6%)
Other	17 (3.2%)	5 (4.0%)	43 (9.9%)
	519	125	435

## Data Availability

No new data were created beyond what is published herein.

## Supplementary Materials

The following supporting information can be downloaded at: https://www.mdpi.com/article/10.3390/diagnostics16030378/s1, Supplementary File S1: Search Strategy; Supplementary File S2: Supplemental methods, including QUADAs; Table S1: Histopathology Table Mapping; Table S2: Excluded Studies; Table S3: Study Characteristics; Figure S1: Summarized Quality Assessment of Risk of Bias Results; Figure S2: Summarized Quality Assessment of Applicability Results; Figure S3: Positivity Rate by Disease Prevalence; Figure S4: Pooled Sensitivity and Specificity with Endometrial Proliferation as Part of Target Condition (Bivariate model); Figure S5: Pooled Miss Rate; Figure S6: PPV and NPV vs. prevalence for fixed sensitivity and specificity; The PRISMA-DTA Checklist.

## Abbreviations

The following abbreviations are used in this manuscript:

BMI	Body Mass Index
CI	Confidence Interval
EEC	Endometrioid Endometrial Carcinoma
EC	Endometrial Cancer
EIN	Endometrial intraepithelial neoplasia
FP	False Positives
FN	False Negatives
HSROC	Hierarchical summary receiver operating characteristic
MPA	Medroxyprogesterone acetate
NPV	Negative predictive value
PCT	Progesterone challenge test
PPV	Positive predictive value
PRISMA-DTA	Preferred Reporting Items for Systematic Reviews and Meta-Analyses of Diagnostic Test Accuracy Studies
QUADAS-2	Quality Assessment of Diagnostic Accuracy Studies tool, version 2
TP	True Positives
TN	True Negatives
