# Supplementary material for: Re-Evaluating the Progesterone Challenge Test as a Physiologic Marker of Endometrial Cancer Risk: A Systematic Review and Meta-Analysis
Source: Diagnostics (Basel). 2026 Jan 23;16(3):378. doi: 10.3390/diagnostics16030378 (PMC12897402; doi:10.3390/diagnostics16030378)
Supplement: Supplementary file 1 [file diagnostics-16-00378-s001.zip › Supplement_Nov25.pdf]

Supplementary File S1: Search Strategy

Medline (Ovid) – Searched 04/10/2025

#	Query
1	menopause/ or postmenopause/
2	(postmenopaus* or post-menopaus*).tw,kf.
3	1 or 2
4	Progesterone/
5	((progesterone* or progestin* or progestogen) adj3 (test* or challenge*)).tw,kf.
6	4 or 5
7	uterine neoplasms/ or endometrial neoplasms/ or carcinoma, endometrioid/
8	((endometrial or endometrium or uterine*) adj4 (cancer* or carcinoma* or neoplasm* or tumo?r* or sarcoma* or malignan* or oncolog*)).tw,kf.
9	7 or 8
10	3 and 6 and 9

Embase (Ovid) – Searched 04/10/2025

#	Query
1	menopause/
2	"menopause and climacterium"/
3	postmenopause/
4	(postmenopaus* or post-menopaus*).tw,kf.
5	1 or 2 or 3 or 4
6	Progesterone/
7	((progesterone* or progestin* or progestogen) adj3 (test* or challenge*)).tw,kf.
8	6 or 7
9	uterus cancer/ or uterus carcinoma/ or uterus tumor/ or uterus sarcoma/ or endometrium cancer/
10	((endometrial or endometrium or uterine*) adj4 (cancer* or carcinoma* or neoplasm* or tumo?r* or sarcoma* or malignan* or oncolog*)).tw,kf.
11	9 or 10
12	5 and 8 and 11

CINAHL (EBSCO) – Searched 04/11/2025

#	Query
S11	S3 AND S6 AND S10
S10	S7 OR S8 OR S9
S9	TI (((endometrial or endometrium or uterine*) N4 (cancer* or carcinoma* or neoplasm* or tumo#r* or sarcoma* or malignan* or oncolog*))) OR AB (((endometrial or endometrium or uterine*) N4 (cancer* or carcinoma* or neoplasm* or tumo#r* or sarcoma* or malignan* or oncolog*)))
S8	(MH "Endometrial Neoplasms")
S7	(MH"Uterine Neoplasms+")
S6	S4 OR S5
S5	TI ((Progesterone* or progestin* or progestogen) N3 (test* or challenge*) OR AB ((Progesterone* or progestin* or progestogen) N3 (test* or challenge*)
S4	(MH "Progesterone+")
S3	S1 OR S
S2	TI ((postmenopaus* or post-menopaus*)) OR AB ((postmenopaus* or post-menopaus*))
S1	(MH "Menopause+")

EBM Reviews (Ovid) – Searched 04/10/2025

Database:
EBM Reviews - Cochrane Database of Systematic Reviews <2005 to January 18, 2023>
EBM Reviews - ACP Journal Club <1991 to December 2022>
EBM Reviews - Database of Abstracts of Reviews of Effects <1st Quarter 2016>
EBM Reviews - Cochrane Clinical Answers <January 2023>
EBM Reviews - Cochrane Central Register of Controlled Trials <December 2022>
EBM Reviews - Cochrane Methodology Register <3rd Quarter 2012>
EBM Reviews - Health Technology Assessment <4th Quarter 2016>
EBM Reviews - NHS Economic Evaluation Database <1st Quarter 2016>

#	Query

1	(postmenopaus* or post-menopaus*).tw,kf.
2	((progesterone* or progestin* or progestogen) adj3 (test* or challenge*)).tw,kf.
3	((endometrial or endometrium or uterine*) adj4 (cancer* or carcinoma* or neoplasm* or tumo?r* or sarcoma* or malignan* or oncolog*)).tw,kf.
4	1 and 2 and 3

Web of Science Search Terms (November 24, 2025)

(TS=(postmenopaus* OR post-menopaus* OR menopause OR postmenopause)) AND (TS=(progesterone OR progestin* OR progestogen* NEAR/3 (test* OR challenge*)) OR TS=(progesterone OR progestin* OR progestogen*)) AND (TS=((endometrial OR endometrium OR uterine*) NEAR/4 (cancer* OR carcinoma* OR neoplasm* OR tumo?r* OR sarcoma* OR malignan* OR oncolog*))OR TS=(uterine neoplasms OR endometrial neoplasms OR endometrioid carcinoma))

Table S1. Histopathology Table Mapping

Category	Histopathology
Benign Conditions	No pathology
	Benign endometrial Polyp/endometrial Polyp
	Benign polypoid
	Submucosal myomas (Fibroids)
	Adenomyosis
	Endometrial tuberculosis
	Senile endometritis
	Endometritis
Atrophic Endometrium	Atrophic Endometrium
	Atrophic with retrogressed cystic hyperplasia
	Inactive endometrium
Endometrial Proliferation	Proliferative Hyper
	Weakly proliferative
	Secretory
Hyperplasia (without atypia)	Simple hyperplasia
	Hyperplasia
	Typical hyperplasia
	Glandular hyperplasia
	Cystic glandular hyperplasia
	Stromal hyperplasia
	Cytoglandular hyperplasia
Atypical Hyperplasia or EIN	Adenomatous hyperplasia

	Atypical adenomatous hyperplasia
	Atypical hyperplasia
**Endometrial Cancer**	Endometrial Adenocarcinoma
	Endometrial Squamous
	Cancer
**Other Pathologies**	Mucus or blood
	Mixed
	Ovarian cancer
	Ovarian thecoma
	Serous ovarian cyst
	Ovarian border-line cystadenoma
	Irregular endometrium

Table S2. Excluded and Included Studies

Citation	Included or excluded	Reason for exclusion
Bortoletto CCR, Baracat EC, Gonçalves WJ, Lima GR, Stávale JN. Transvaginal ultrasonography and the progestogen challenge test in postmenopausal endometrial evaluation. International Journal of Gynecology & Obstetrics. 1997 Sep 16;58(3):293–8.	Excluded	Duplicate results to already included article
Dowling EA, Gravlee LC, Hutchins KE. A new technique for the detection of adenocarcinoma of the endometrium.	Excluded	Wrong outcome – assessment of endometrial sampling tool

Acta cytologica. 1969 Sep;13(9):496–501.		
Gambrell RD. The prevention of endometrial cancer in postmenopausal women with progestogens. Maturitas. 1978 Sep;1(2):107–12.	Excluded	Wrong outcome - incidence of EC in patients, effect of progestogen treatments, number of patients with cancer
Gambrell RD, Castaneda TA, Ricci CA. Management of postmenopausal bleeding to prevent endometrial cancer. Maturitas. 1978 Sep;1(2):99–106.	Excluded	Wrong outcome – assessing pathology and abnormal uterine bleeding
Gambrell RD, Massey FM, Castaneda TA, Ugenas AJ, Ricci CA. Reduced Incidence of Endometrial Cancer among Postmenopausal Women Treated with Progestogens*. Journal	Excluded	Wrong outcome – investigating incidence of endometrial cancer

of the American Geriatrics Society. 1979 Sep 27;27(9):389–94.		
Gambrell RD, Massey FM, Castaneda TA, Ugenas AJ, Ricci CA, Wright JM. Use of the progestogen challenge test to reduce the risk of endometrial cancer. Obstetrics and gynecology. 1980 Jun;55(6):732–8.	Excluded	Wrong outcome – does not report total progesterone challenge tests administered and proportion of positive test results
Don Gambrell R. Role of Hormones in the Etiology and Prevention of Endometrial and Breast Cancer. Acta Obstetricia et Gynecologica Scandinavica. 1982 Jan 11;61(S106):37–46.	Excluded	Wrong outcome - incidence of breast cancer and endometrial cancer in postmenopausal individuals on various hormonal regimens
Whitehead M, Siddle N, Townsend P, Lane G, King R. The use of progestins and progesterone in the treatment of climacteric and postmenopausal	Excluded	Wrong outcome – investigating various types of progestogens

symptoms. Progesterone and progestins. 1983;277–94.		
Gambrell RD. Cancer and the use of estrogens. International journal of fertility. 1986;31(2):112–3, 116–22.	Excluded	Wrong outcome – incidence of endometrial cancer and breast cancer in postmenopausal individuals on hormone replacement therapy compared to untreated
Gambrell RD. Prevention of endometrial cancer with progestogens. Maturitas. 1986 Jul;8(2):159–68.	Excluded	Wrong outcome – investigating incidence of endometrial cancer
Gorodeski IG, Geier A, Lunenfeld B, Bahary CM. Progesterone Challenge Test in Postmenopausal Women with Pathological Endometrium. Cancer Investigation. 1988 Jan 11;6(5):481–5.	Excluded	Wrong outcome - assessing progesterone impact on progesterone receptor levels in endometrial tissue

NASRI MN, SHEPHERD JH, SETCHELL ME, LOWE DG, CHARD T. The role of vaginal scan in measurement of endometrial thickness in postmenopausal women. BJOG: An International Journal of Obstetrics & Gynaecology. 1991 May 19;98(5):470–5.	Excluded	Wrong outcome - assessing transvaginal sonography and histopathology findings
de Cicco F, Valenzano A, Rossiello F, Trecca P, Cinque B, Mango D, et al. Medroxy-progesterone acetate test (MPA-test) for screening of endometrial adenocarcinoma in asymptomatic post-menopausal women . Acia med rom. 1994;32:538–43.	Excluded	Wrong outcome – does not report Progesterone Challenge Test positive test proportion or overall tests administered
Gillet JY, Andre G, Faguer B, Erny R, Buvat-Herbaut M, Domin MA, et al. Induction of amenorrhea during	Excluded	Wrong outcome - assessment of specific micronized progesterone on endometrium

hormone replacement therapy: Optimal micronized progesterone dose. A multicenter study. Maturitas. 1994 Aug;19(2):103–15.		
Meuwissen JHJM, van Langen H, Navarro I. Ultrasound determination of the effect of progestogens on the endometrium in postmenopausal women receiving hormone replacement therapy. Maturitas. 1994 Feb;18(2):77–85.	Excluded	Wrong outcome - assessed endometrial thickness changes in response to progestogen addition to hormone replacement therapy
Meuwissen JHJM, Wiegerinck MAHM, Haverkorn MJ. Regression of endometrial thickness in combination with reduced withdrawal bleeding as a progestational effect of tibolone in postmenopausal women on oestrogen replacment therapy. Maturitas. 1995 Feb;21(2):121–5.	Excluded	Wrong outcome – investigating effects of progesterone on endometrium in postmenopausal individuals on estrogen therapy

Morcos RN, Denice Leonard M, Smith M, Bourguet C, Makii M, Khawli O. Vaginosonographic measurement of endometrial thickness in the evaluation of amenorrhea. Fertility and Sterility. 1991 Mar;55(3):543–6.	Excluded	Wrong patient population - population with amenorrhea
NAKAMURA S, DOUCHI T, OKI T, IJUIN H, YAMAMOTO S, NAGATA Y. Relationship between sonographic endometrial thickness and progestin-induced withdrawal bleeding. Obstetrics & Gynecology. 1996 May;87:722–5.	Excluded	Wrong patient population – population with secondary amenorrhea
Panay N, Pritsch M, Alt J. Cyclical dydrogesterone in secondary amenorrhea: Results of a double-blind, placebo-controlled, randomized study.	Excluded	Wrong patient population - premenopausal individuals with secondary amenorrhea

Gynecological Endocrinology. 2007 Jan 7;23(11):611–8.		
Hempel E, Nöschel H, Eichhorn KH, Rasch A, Franke D. [Comparison of the progesterone test and uterus sonography as screening procedures in the detection of patients at risk of endometrial cancer]. Zentralblatt fur Gynakologie. 1988;110(10):597–602.	Excluded	Not published in English or French
Kawabata M, Tsuda H, Kawabata K, Umesaki N, Yamagata S, Sugawa T. [Ultrasonographic evaluation of the endometrium in postmenopausal women to select the high risk group of endometrial carcinoma of the uterus]. Nihon Sanka Fujinka Gakkai zasshi. 1989 Oct;41(10):1512–6.	Excluded	Not published in English or French

Ivanov S, Karag'ozov A. [The progesterone test and uterine sonography as screening methods in women during the postmenopause with and without diabetes mellitus]. Akusherstvo i ginekologiia. 1991;30(1):52–5.	Excluded	Not published in English or French
Ivanov S, Karag'ozov A, Kŭrlov T, Diankova T, Gancheva A, Chakalova G. [The early detection and screening of endometrial carcinoma by the progesterone test and uterine sonography]. Akusherstvo i ginekologiia. 1993;32(2):24–5.	Excluded	Not published in English or French
Valenzuela P, Nieto A. Progesterone Stimulation test and obesity. Maturitas . 1995;21(1):77.	Excluded	Not published in English or French

Valenzuela P, Nieto A. Prueba de estimulación con progesterona y niveles hormonales circulantes . Clin Invest Gin Obst . 1993;20(10):434–6.	Excluded	Not published in English or French
Ivanov S, Karag'ozov A, Kŭrlov T, Grueva A, Karag'ozov I. [A comparative study of the potentials of a cytological method, the progesterone test and echography in the early detection of endometrial carcinoma in women in the postmenopause]. Akusherstvo i ginekologiia. 1994;33(2):31–2.	Excluded	Not published in English or French
Ivanov S. [Uterine sonography as a method for the early detection and screening of endometrial carcinoma in women in the postmenopause with and without diabetes mellitus]. Akusherstvo i ginekologiia. 1994;33(3):21–2.	Excluded	Not published in English or French

Valenzuela P, Nieto A. Progesterone Stimulation test and obesity. Maturitas . 1995;21(1):77.	Excluded	Not published in English or French
Mele GA, Pirosu T, Orru MC. [Early diagnosis of endometrial carcinoma and its precursors in asymptomatic postmenopausal women. Proposal of a diagnostic protocol]. Minerva ginecologica. 1997 Apr;49(4):133–7.	Excluded	Not published in English or French
Ivanov S, Ivanov S. [The progesterone test and transvaginal sonography as methods for the early discovery and screening of endometrial cancer in women in the postmenopause from some risk groups]. Akusherstvo i ginekologiia. 1999;38(2):29–30.	Excluded	Not published in English or French

Ivanov S. [The use of hysterosonography and transvaginal sonography in combination with the progesterone test in asymptomatic women in risk groups in the postmenopause]. Akusherstvo i ginekologiia. 1999;38(4):18–20.	Excluded	Not published in English or French
Wang Y kui, Zhang S miao, Xu B. PROGESTERONE CHALLENGE TEST AND TRANSVAGINAL ULTRASONOGRAPHY IN THE DETECTION OF ENDOMETRIAL PATHOLOGY IN POSTMENOPAUSAL WOMEN. QILU MEDICAL JOURNAL. 2004;19(4):287–90.	Excluded	Not published in English or French
BASIRAT Z, ESMAEILZADEH S, HASAS DALIRKHAH F. PROGESTERONE CHALLENGE TEST FOR DIAGNOSIS ENDOMETRIUM IN 50 MENOPAUSAL	Excluded	Not published in English or French

WOMEN OF PROLIFERATIVE . JOURNAL OF BABOL UNIVERSITY OF MEDICAL SCIENCES (JBUMS) . 2007;8(6):30–4.		
Studd JWW, Thom MH, Paterson MEL, Wade-Evans T. The Prevention and Treatment of Endometrial Pathology in Postmenopausal Women receiving Exogenous Estrogens. In: The Menopause and Postmenopause. Dordrecht: Springer Netherlands; 1980. p. 127–39.	Excluded	Not a primary research article – book chapter/clinical guide
Gambrell R. Preventing endometrial Ca with progestin. Contemporary OBGYN. 1981;17:133–42.	Excluded	Not a primary research article – clinical review
Kantor HI. Progestogen Challenge Test. Obstetrics & Gynecology [Internet]. 1981;57(2). Available from:	Excluded	Not a primary research article – letter to editor

https://journals.lww.com/greenjournal/fulltext/1981/02000/progestogen_challenge_test.30.aspx		
Gambrell RD. Clinical use of progestins in the menopausal patient: dosage and duration. The Journal of reproductive medicine. 1982 Aug;27(8 Suppl):531–8.	Excluded	Not a primary research article - clinical review/management recommendation
Greenblatt RB, Gambrell RD, Stoddard LD. The protective role of progesterone in the prevention of endometrial cancer. Pathology, research and practice. 1982 Aug;174(3):297–318.	Excluded	Not a primary research article – clinical review
Whitehead MI, Townsend PT, Pryse-Davies J, Ryder T, Lane G, Siddle NC, et al. Effects of various types and dosages of progestogens on the	Excluded	Not a primary research article – clinical review

postmenopausal endometrium. The Journal of reproductive medicine. 1982 Aug;27(8 Suppl):539–48.		
Hoff J, Poulhes J. [Cancer of the endometrium: detection and prophylaxis]. La semaine des hopitaux : organe fonde par l’Association d’enseignement medical des hopitaux de Paris. 1983 Jan 20;59(3):198–200.	Excluded	Not a primary research article - clinical review
Gambrell RD. The role of hormones in the etiology and prevention of endometrial cancer. Clinics in obstetrics and gynaecology. 1986 Dec;13(4):695–723.	Excluded	Not a primary research article - clinical review/management recommendation
Gambrell RD. Use of progestogen therapy. American journal of	Excluded	Not a primary research article – clinical review

obstetrics and gynecology. 1987 May;156(5):1304–13.		
Gambrell RD. Cancer in the older woman: diagnosis and prevention. Geriatrics. 1988 Sep;43(9):27–32, 35–6.	Excluded	Not a primary research article – clinical review
Gambrell R. Progestogens and Postmenopausal Women. The Female Patient. 1992;17:33–52.	Excluded	Not a primary research article - clinical review/management recommendation
Gambrell RD. Progestogens in estrogen-replacement therapy. Clinical obstetrics and gynecology. 1995 Dec;38(4):890–901.	Excluded	Not a primary research article – clinical review
Kable PWT, Bewtra C, Gallagher JC. Use of Progestin Challenge Test in	Excluded	Not a primary research article – conference abstract

Elderly Women. Menopause. 1995;2(4):278.		
Gambrell Jr. RD. Strategies to reduce the incidence of endometrial cancer in postmenopausal women. American Journal of Obstetrics & Gynecology [Internet]. 1997 Nov 1;177(5):1196–207. Available from: https://doi.org/10.1016/S0002-9378(97)70040-1	Excluded	Not a primary research article – literature review
Rice L. Tamoxifen and endometrial cancer screening. MENOPAUSE-THE JOURNAL OF THE NORTH AMERICAN MENOPAUSE SOCIETY. 1997;4(1):3–5.	Excluded	Not a primary research article – editorial
Chu H. The value of progesterone challenge test combined with transvaginal ultrasonography detect endometrial diseases in postmenopausal asymptomatic women. Ultrasound in Medicine & Biology. 2006 May;32(5):P235.	Excluded	Not a primary research article – conference abstract

Römer T. Hormone replacement therapy and bleeding disorders. Gynecological endocrinology : the official journal of the International Society of Gynecological Endocrinology. 2006 Mar;22(3):140–4.	Excluded	Not a primary research article - clinical review/management recommendation
Archer DF. A simple inexpensive screening test for endometrial abnormalities in asymptomatic women using tamoxifen. Menopause. 2010;17(3):454–5.	Excluded	Not a primary research article – editorial
Erny R. Screening of endometrial carcinoma. In Difusion Vigot; 1987. p. 5.	Excluded	No access
Pascual MA. Progesterone challenge test in menopausal women. Clin Invest Gynecol Obstet. 1987;14:130.	Excluded	No access

Wehba S. Progesterone test for detection of endometrium hyperplastic lesions in postmenopausal women. [São Paulo]; 1988.	Excluded	No access
Hanna JH, Brady WK, Hill JM, Phillips Jr. GL. Detection of postmenopausal women at risk for endometrial carcinoma by a progesterone challenge test. American Journal of Obstetrics & Gynecology [Internet]. 1983 Dec 15;147(8):872–5. Available from: https://doi.org/10.1016/0002-9378(83)90238-7	Included	Not applicable
Erny R, Serradimigni F. Dépistage des hyperplasies et des cancers de l'endomètre. Rev fr Gynécol Obstét. 1984;79(2):91–6.	Included	Not applicable
Erny R, Isnard S, Boubli L. [Progestogen tests after the menopause]. Revue francaise de gynecologie et d'obstetrique. 1986 Apr;81(4):195–8.	Included	Not applicable
Toppozada MK, Ismail AAA, Hamed RSM, Sid Ahmed K, EL-Faras A. Progesterone challenge test and estrogen assays in menopausal women with endometrial adenomatous hyperplasia. International Journal of Gynecology & Obstetrics [Internet]. 1988 Feb	Included	Not applicable

1;26(1):115–9. Available from: https://doi.org/10.1016/0020-7292(88)90205-6		
Viel A, Goriaux J, Rouge J, Crouet H, Héron J. Le test à la progestérone est-il la méthode généralisable de dépistage des lésions néoplasiques et prénéoplasiques de l'endomètre? Bull Cancer . 1990;77:371–6.	Included	Not applicable
Pansini F, de Paoli D, Serra MM, Campobasso C, Levato F, Giulini D. Combined use of progesterone challenge test and endometrium thickness evaluated by transvaginal ultrasonography in the preventive management of postmenopausal women. Gynecologic and obstetric investigation. 1992;34(4):237–9.	Included	Not applicable
Valenzuela P, Sabatel RM, Valls V, Nieto A, Gonzalez-Gomez F. Progestin challenge test in postmenopausal patients. International journal of gynaecology and obstetrics: the official organ of the International Federation of Gynaecology and Obstetrics. 1993 Dec;43(3):313–6.	Included	Not applicable
Macia M, Novo A, Ces J, González M, Quintana S, Codesido J. Progesterone challenge test for the assessment of endometrial pathology in asymptomatic menopausal women. International Journal of Gynecology & Obstetrics [Internet]. 1993 Feb 1;40(2):145–9. Available from: https://doi.org/10.1016/0020-7292(93)90375-7	Included	Not applicable
Deka D, Buckshee K. Progesterone Challenge Test in Screening	Included	Not applicable

Asymptomatic Postmenopausal Women for Cancer Endometrium . Journal of Obstetrics and Gynaecology of India. 1995 Oct 20;746–9.		
Bortoletto C de C, Baracat EC, Gonçalves WJ, Stávalle JN, de Lima GR, Novo NF. The progestogen challenge test in postmenopausal women: clinical and morphologic aspects. Sao Paulo medical journal = Revista paulista de medicina. 1996;114(3):1166–72.	Included	Not applicable
Pansini F, de Paoli D, Albertazzi P, Bonaccorsi G, Campobasso C, Zanotti L, et al. Sequential addition of low dose of medrogestone or medroxyprogesterone acetate to transdermal estradiol: a pilot study on their influence on the endometrium. European journal of obstetrics, gynecology, and reproductive biology. 1996 Sep;68(1–2):137–41.	Included	Not applicable
Büyük E, Durmuşoğlu F, Dökmeci C. Effect of initial gestagen treatment on bleeding patterns in postmenopausal women receiving continuous combined hormone replacement therapy. Menopause (New York, NY). 1999;6(2):156–60.	Included	Not applicable
Madhuri K. Progesterone Challenge Test in Screening Postmenopausal Women for Risk of Endometrial Carcinoma a Comparative Study with Endometrial Thickness by Transvaginal Sonography . [Vijayawada]; 2001.	Included	Not applicable
El-Maraghy MA, El-Badawy N, Wafa GA, Bishai N. Progesterone challenge test in postmenopausal women at high	Included	Not applicable

risk. Maturitas [Internet]. 1994 May 1;19(1):53–7. Available from: https://doi.org/10.1016/0378-5122(94)90041-8		
Malinova M, Pehlivanov B. Transvaginal sonography and progesterone challenge for identifying endometrial pathology in postmenopausal women. International Journal of Gynecology & Obstetrics [Internet]. 1996 Jan 1;52(1):49–53. Available from: https://doi.org/10.1016/0020-7292(95)02554-5	Included	Not applicable
Guerrieri JP, Elkas JC, Nash JD. Evaluating the Endometrium in Women on Tamoxifen: A Pilot Study to Compare a "Gold Standard" with an "Old Standard." Menopause [Internet]. 1997;4(1). Available from: https://journals.lww.com/menopausejournal/Fulltext/1997/04010/Evaluating_the_Endometrium_in_Women_on_Tamoxifen__.3.aspx	Included	Not applicable
Pehlivanov B, Malinova M, Grozdanov G. Progesterone challenge test for evaluation of endometrial hyperplasia in postmenopausal women. Folia medica. 1998;40(2):22–5.	Included	Not applicable
Rani R, Devi K, Papa D, Jayanthi S. Transvaginal Sonography and Progesterone Challenge Test for Identifying Endometrial Pathology in Post Menopausal Women. Journal of Obstetrics and Gynaecology of India. 2002 Jan;52(1):135–8.	Included	Not applicable
Lubian López DM, Fernandez YG, Rodríguez BR, López FMO, Delgado RC.	Included	Not applicable

Value of the progesterone test in screening for endometrial pathology in asymptomatic postmenopausal women receiving treatment with tamoxifen. Menopause (New York, NY). 2010 May;17(3):487–93.		

Supplementary File S2. Supplemental Methods

Supplementary Methods- QUADAS2 Tool Development and Navigation

The QUADAS 2 tool investigates the risk of bias across four domains including patient selection, index test, reference standards and the flow of participants in the study. This tool is appropriate to report bias and assess applicability in diagnostic test evaluation studies [26].

Phase 1: Review Question

Patients of interest: asymptomatic postmenopausal women undergoing the Progesterone Challenge Test (PCT).

The index test: any short course of a synthetic progestogen or progesterone administered for any time, followed by surveillance for abnormal withdrawal bleeding after discontinuation of the regimen.

The reference standard: an endometrial biopsy.

The target condition: any endometrial pathology, including endometrial hyperplasia, adenomatous hyperplasia, endometrial intraepithelial neoplasia (EIN) and endometrial carcinoma. Endometrial proliferation is not considered a pathology, but is indicative of the presence of unopposed estrogen in postmenopause, which may lead to pathology. In our

review, we did two separate analyses where proliferation was considered as part of, or excluded from the target condition.

The review question: The diagnostic accuracy of the PCT to identify asymptomatic post-menopausal women who are at increased risk for endometrial pathology.

Phase 2: Tool Tailoring to the Review

To tailor the QUADAS-2 tool, two additional signaling questions were added to the existing questions.

1) "Did the PCT definition include dosage, timing, withdrawal monitoring window and bleeding as a result?" (domain 2).
2) "Did all patients receive the same index test" (domain 4).

To tailor the QUADAS-2 tool, two signalling questions were removed. One was from the second domain and was removed to reduce redundancy with a signalling question the authors added in the previous step. The second signalling question was removed from domain 4 since studies not administering a reference standard was not a concern as studies which did not were excluded from analysis requiring reference standard results in this study.

1) "If a threshold was used, was it pre-specified?" (domain 2)
2) "Did all patients receive a reference standard?" (domain 4)

Phase 3: Flow Chart of Study Design

A flow chart was drawn for each study to represent study and participant flow. If a flow chart was included in the paper, it was compared to the drawn flow chart and any discrepancies were reassessed.

Phase 4: Judging Bias and Applicability of Studies

Bias and applicability were assessed in four domains: patient selection, index test, reference standard and flow and timing. The risk of bias is first assessed in each domain through signaling questions that can be answered “yes”, “no”, or “unclear”. “Yes” is indicative of low bias, whereas “no” responses suggest there may be some concern for bias for that signaling question. If any signaling question in a domain is answered “no”, then there is risk for bias and authors would meet to discuss the risk of bias using the outlines developed in phase 2 [26]. “Unclear” was only used if the answer to the signaling question was not reported in the paper. When all signaling questions are answered “yes” for a domain, then there is a low risk of bias [26]. When there was "yes" responses and “unclear” responses, the authors navigating the tool would indicate that domain as being overall “unclear” since simply stating “low risk” due to the presence of “yes” responses and absence of “no” responses could be misleading, as omission of information which warranted the “unclear” response could be indicative of bias present.

Signaling questions:

Domain 1: PATIENT SELECTION

Part A: Risk of Bias

1) Was a consecutive or random sample of patients enrolled?

2) Was a case-control design avoided?
3) Did the study avoid inappropriate exclusions?

1A. Could the selection of patients have introduced bias?

Part B: Applicability

1B. Is there concern that the included patients do not match the review question?

Domain 2: INDEX TEST

1) Did the PCT definition include dosage, timing, withdrawal monitoring window and bleeding as a result?*
2) Were the index test results interpreted without knowledge of the results of the reference standard?

2A. Could the conduct or interpretation of the index test have introduced bias?

2B. Is there concern that the index test, its conduct, or interpretation differ from the review question?

Domain 3: REFERENCE STANDARD

1) Is the reference standard likely to correctly classify the target condition?
2) Were the reference standard results interpreted without knowledge of the results of the index test?

3A: Could the reference standard, its conduct, or its interpretation have introduced bias?

3B: Is there concern that the target condition as defined by the reference standard does not match the review question?

Domain 4: FLOW AND TIMING

1) Was there an appropriate interval between index test and reference standard?
2) Did patients receive the same reference standard?
3) Did all patients receive the same index test?*
4) Were all patients included in the analysis?

4A: Could the patient flow have introduced bias?

*Signalling questions added in Phase 2

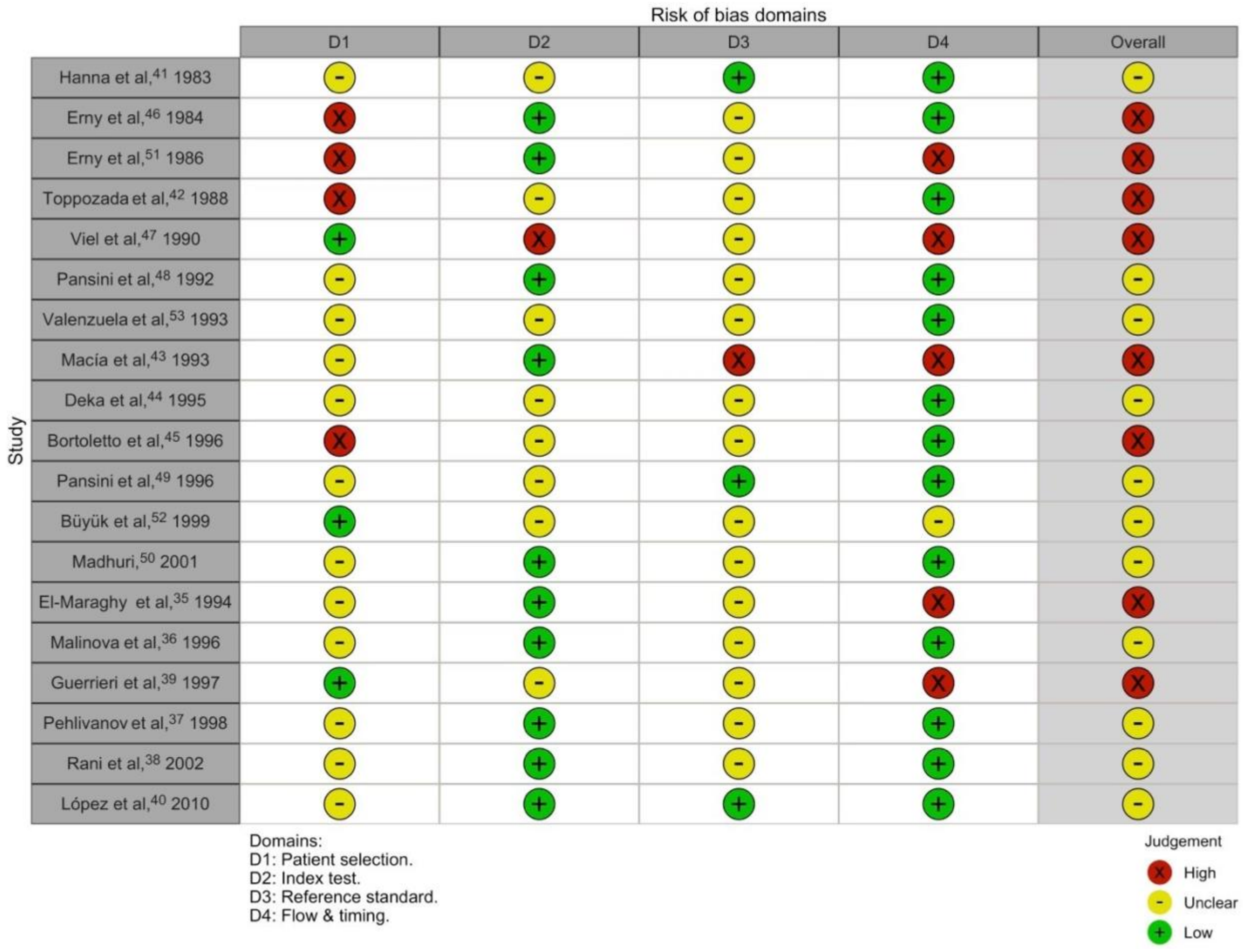

Figure S1. Summarized Quality Assessment of Risk of Bias Results

The traffic light plot summarizes individual study risk of bias for each of the four domains (D1-D4). The red circle indicates a high risk of bias, the yellow circle indicates an unclear risk of bias, and the green circle represents a low risk of bias for that domain. The overall risk of bias among all domains for each study is summarized in the “overall” column.

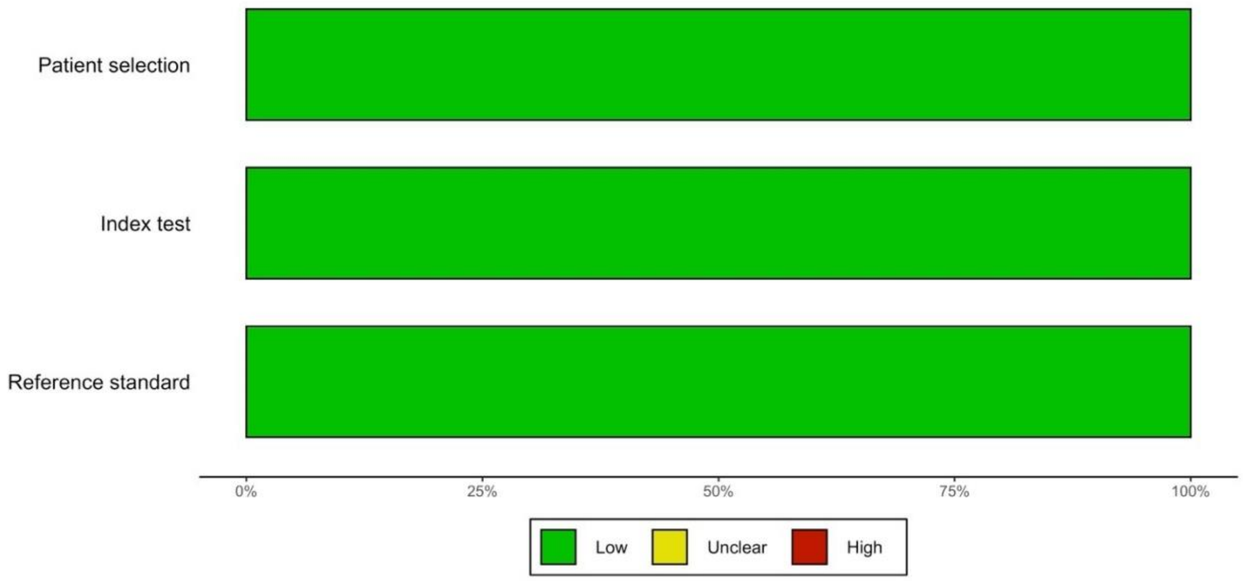

Figure S2. Summarized Quality Assessment of Applicability Results

Summary of applicability for three domains (D1-D3). Green indicates low risk for applicability, yellow indicates unclear risk of applicability and red is high-risk of study applicability summarized for each domain.

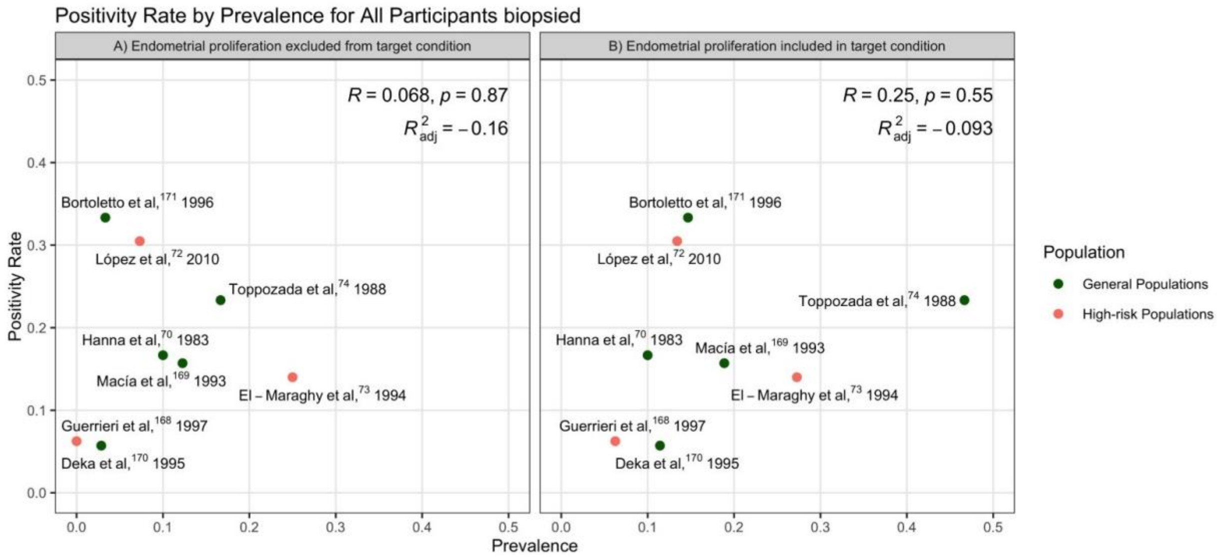

Figure S3. Positivity Rate by Disease Prevalence

Positivity rate of individual studies by prevalence for both excluding endometrial proliferation from target condition (left) and including (right). Abbreviations: R = Pearson correlation coefficient; R^2_{adj} = Corrected goodness-of-fit measure.

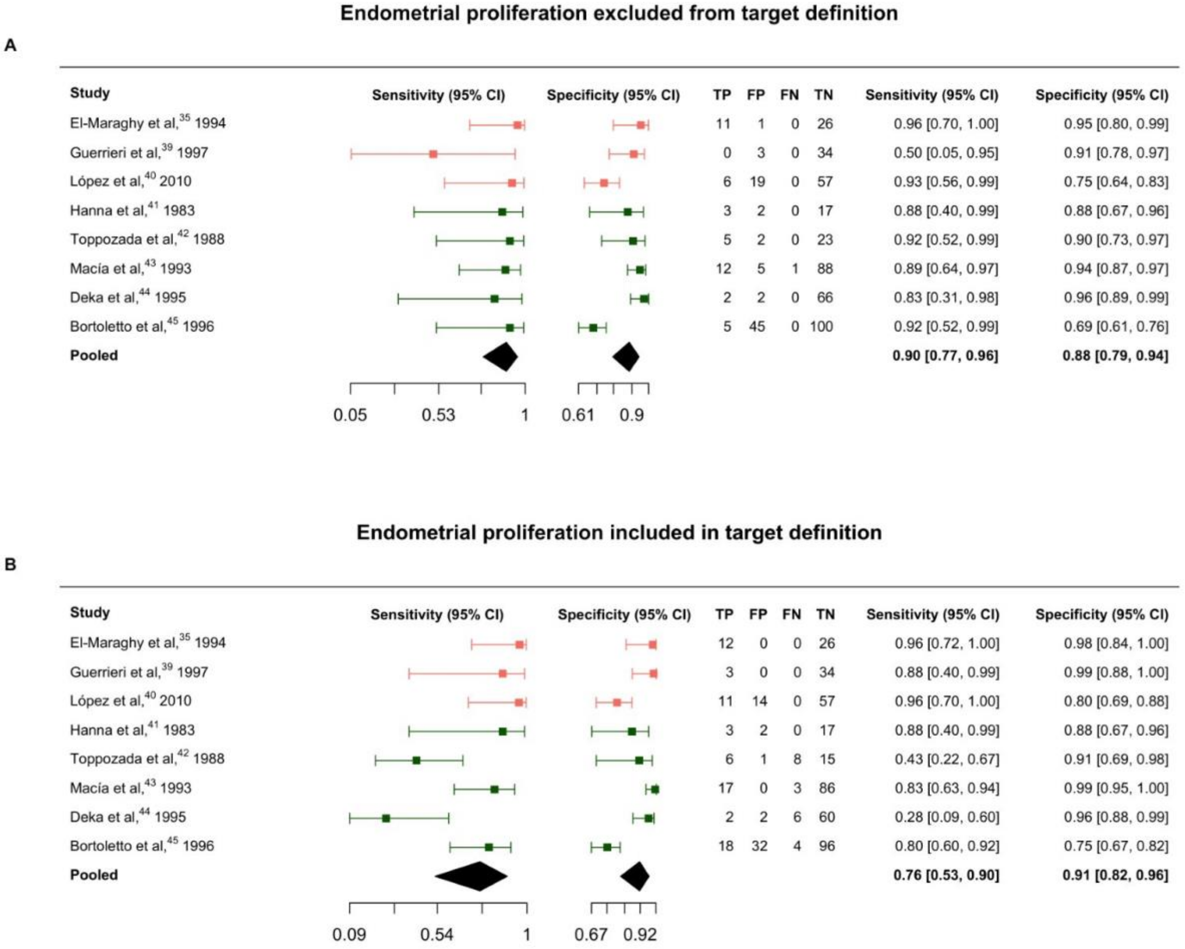

Figure S4. Pooled Sensitivity and Specificity with Endometrial Proliferation as Part of Target Condition (Bivariate model)

A) Target condition excludes endometrial proliferation and B) Target condition includes endometrial proliferation. High-risk populations are highlighted in dark green. General populations are highlighted in salmon pink. Abbreviations: CI = Confidence Interval; TP = True Positive; FP = False Positive; FN = False Negative; TN = True Negative.

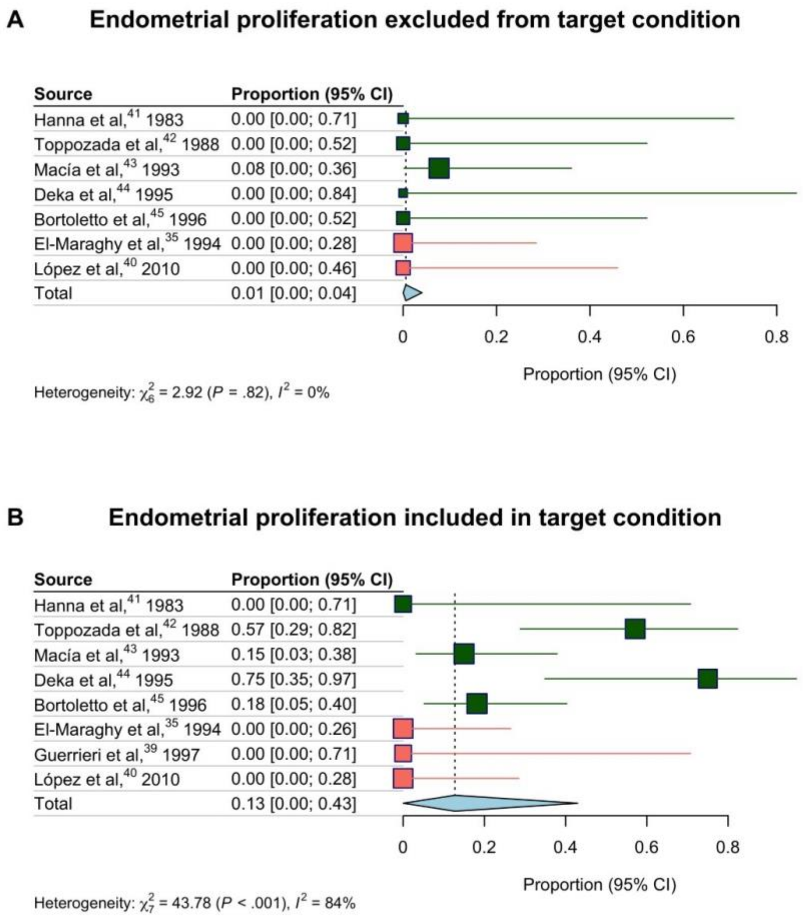

Figure S5. Pooled Miss Rate

A) Target condition excludes endometrial proliferation and B) Target condition includes endometrial proliferation. High-risk populations are highlighted in dark green. General populations are highlighted in salmon pink. Abbreviations: CI = Confidence Interval.

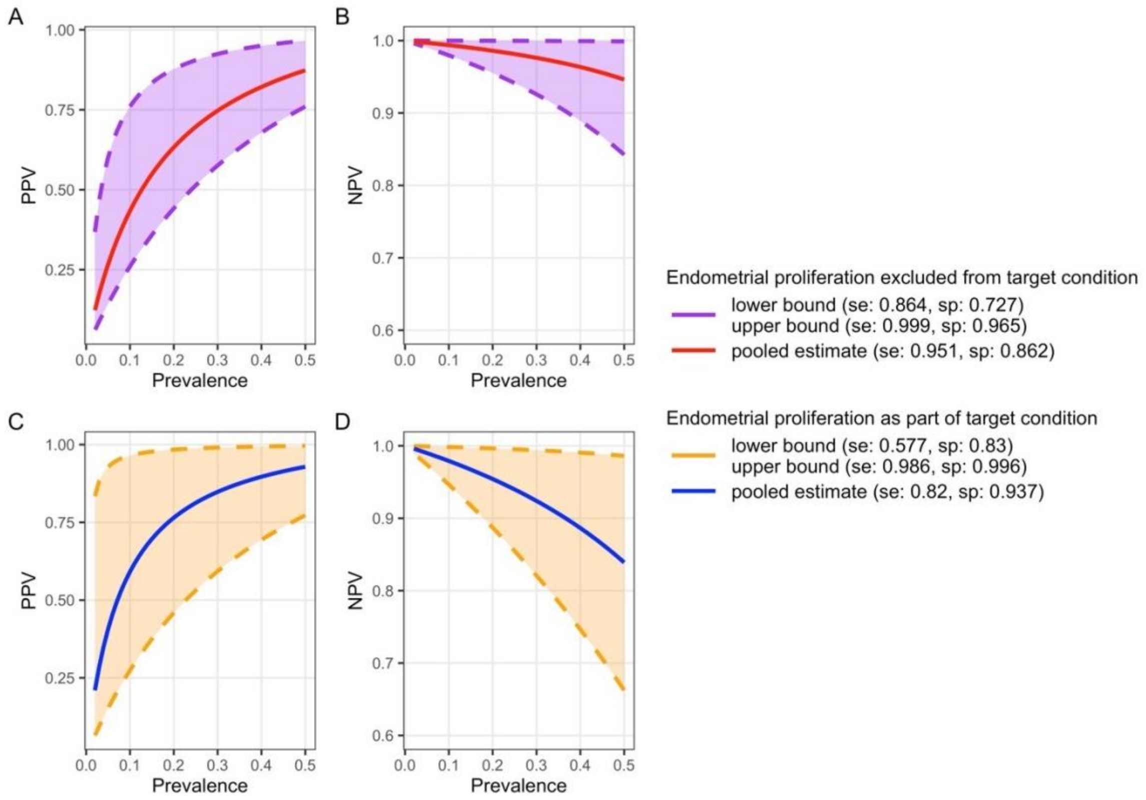

Figure S6. PPV and NPV vs. prevalence for fixed sensitivity and specificity

A: Proliferative excluded from target condition, PPV; B: Proliferative excluded from target condition, NPV; C: Proliferative as part of target condition, PPV; D: Proliferative as part of target condition, NPV

Abbreviations: PPV = Positive Predictive Value; NPV = Negative Predictive Value; se = Sensitivity; sp = Specificity
